# Drug Development Using Natural Toxins

**DOI:** 10.3390/toxins13060414

**Published:** 2021-06-11

**Authors:** Gihyun Lee

**Affiliations:** College of Korean Medicine, Dongshin University, Naju 58245, Korea; glee@khu.ac.kr

Natural toxins are poisonous substances produced by bacteria, insects, animals, or plants. They cause pain, disease, and even death to victims, but they also provide humans with a rich resource for new drugs for a variety of targets, from pain to lethal diseases [1]. There are successful examples of venom-based drug such as Prialt^®^ and Exenatide^®^ [2].

However, most toxins have not been studied enough as drug candidates, even though they have long been used to treat diseases globally. Bee venom, one of the most widely used toxins used for therapeutic purposes, is still being studied for its efficacy and safety as a remedy for many diseases [3]. Other poisons such as those from snake, scorpion, and toad venoms are at the beginning stage of drug development, as well as toxic medicinal plants.

In this Special Issue, “Drug Development Using Natural toxins”, we have attempted to provide the reader with a comprehensive overview of natural toxins with therapeutic potential. This issue focuses on the mechanism of action, drug development, adverse effects, and clinical applicability including, but not limited to, novel developments relating to venoms with clinical potential. The following is a short synopsis of the six reviews and six research papers that constitute this Special Issue.

A study by Ryan et al. [4] investigated the immune suppressive potential of red-bellied black snake venom. They showed that the venom blocked secretion of IL-2 and TNF from human T cells but not IL-6 or TNF release from antigen-presenting cells. The reduced cytokine release from T cells was not interrelated with blocking of T cell proliferation or reduction of cell viability, consistent with an anti-inflammatory mechanism unrelated to the cell cycle.

Three papers deal with the antibacterial activity of natural toxins. Zhou et al. [5] demonstrated novel antimicrobial peptides (AMPs) from the skin secretion of *Odorrana schmackeri*. They designed the analogs by altering the key factors, including amphipathicity, conformation, and net charge to generate short AMPs with enhanced therapeutic efficacy. The authors suggested that a balance between positive charge, degrees of α-helicity, and hydrophobicity is necessary for maintaining antimicrobial activity, and these data successfully contributed to the design of short AMPs with significant bactericidal activity and cell selectivity. Sam Woong Kim et al. [6] reported that AMPs secreted from *Lactobacillus taiwanensis* effectively form ghosts of pathogenic bacteria and are identified as diverse bacteriocins, including novel bacteriocins. Their research results suggest that the bacteriocins from *L. taiwanensis* are potentially useful as a critical constituent for the preparation of bacterial ghosts. Esteban et al. [7] studied the antibacterial activity of gliotoxin alone and in combination with antibiotics against *Staphylococcus aureus* in vitro and in vivo. They showed that gliotoxin presents antimicrobial activity in vitro and in vivo against a clinically relevant methicillin-resistant *Staphylococcus aureus* and vancomycin-intermediate *S. aureus* strain. The authors suggested that the ability of gliotoxin to synergistically enhance the effect of vancomycin presents promising prospects for new drug development to help in treating drug-resistant strains and to reduce the emergence of vancomycin-resistant strains.

Kuna et al. [8] evaluated the antifungal activity of *Naja pallida* and *Naja mossambica* venoms against three *Candida* species. They characterized the proteomes of the venoms of *Naja pallida* and *Naja mossambica* and analyzed the antifungal properties of crude venoms against three *Candida* species, namely *C. albicans*, *C. glabrata*, and *C. tropicalis*. A complex response to *Naja pallida* and *Naja mossambica* venom treatments was revealed. They showed that the venom of *Naja pallida* and *Naja mossambica* also stimulated the secretion of extracellular phospholipases that may facilitate Candida pathogenicity and limit their applications as anticandidal agents.

The paper by Wonnam Kim et al. [9] explored the anti-inflammatory effect of *Ephedra sinica* Stapf in a heat-induced mouse model. Though *Ephedra sinica* Stapf exerts toxic effects, such as tremor, excitability, headache, anxiety, insomnia, nausea, vomiting, poor appetite, cardiac arrhythmia, convulsions, and others [10], it is used most often to treat symptoms caused by external stressors in traditional herbal medicine. The authors showed that *Ephedra sinica* Stapf treatment ameliorated body weight loss, increased body temperature, and HSP70 and NF-kB changes by heat stress. Their results suggest that *Ephedra sinica* Stapf contributes to the inhibition of heat-induced proinflammatory factors and the promotion of hypothalamic homeostasis.

Two papers within this Special Issue are focused on bee venom. Jasmin Katrin Badawi’s review paper [11] describes bee venom constituents as therapeutic tools against prostate cancer. The paper summarizes different approaches using bee venom, melittin, modified melittin, or a protoxin as anticancer agents. The toxic agents acted through several different mechanisms to produce their antiprostate cancer effects. Jang et al. [12] reviewed the clinical effectiveness of bee venom and adverse events induced by bee venom, regardless of the disease, using four electronic databases in April 2020. Twelve randomized controlled trials were included in the review—four on arthralgia, four on musculoskeletal disorders, three on Parkinson’s disease, and one on polycystic ovary syndrome. Six studies reported adverse events, and skin reactions such as pruritus and swelling were the most common.

Hwang et al. [13] explored studies using toxic animal-based medicinal materials as an endometriosis treatment and explored their clinical applicability. Preclinical and clinical studies using toxic animal-based medicinal materials (TMMs) were searched for in four databases from inception to October 2020. A total of twenty studies on TMMs for endometriosis were included. The main therapeutic mechanisms of TMMs against endometriosis were antiangiogenesis, estrogen level reductions, proapoptotic effects, and possible anti-inflammatory effects in twelve experimental studies. In eight clinical studies, herbal medicines containing TMMs were effective in relieving symptoms of endometriosis with no adverse effects. 

Sung et al. [14] investigated the use of animal venom pharmacopuncture in Korean medicine institutions to contribute to the development of animal venom-based medicines. They surveyed 256 public health centers from 1 through 31 October 2019 as guided by the Ministry of Health and Welfare of Korea. The survey identified three types of animal venom-based pharmacopuncture: bee, toad, and snake venoms. Their study shows that bee, toad, and snake venoms could be used in medical institutions and have the potential for drug development.

Heli Sätilä [15] studied over twenty-five years of pediatric botulinum toxin treatments. Her study reviewed botulinum toxin type A injection techniques, doses and dilutions, the recovery of muscles, and the impact of repeated injections, with a focus on the pediatric population.

Lastly, the paper by Gremski et al. [16] reviewed forty years of the description of phospholipase D from *Loxosceles* venoms. It presents information gleaned over the last forty years about brown spider venom phospholipase D, especially with regard to the production and characterization of recombinant isoforms. The history of obtaining these toxins is discussed, as well as their molecular organization and mechanisms of interaction with their substrates. The authors discussed cellular biology aspects of these toxins and how they can be used in the development of drugs to address inflammatory processes and loxoscelism.
